# Angle-dependent magnetotransport in GaAs/InAs core/shell nanowires

**DOI:** 10.1038/srep24573

**Published:** 2016-04-19

**Authors:** Fabian Haas, Tobias Wenz, Patrick Zellekens, Nataliya Demarina, Torsten Rieger, Mihail Lepsa, Detlev Grützmacher, Hans Lüth, Thomas Schäpers

**Affiliations:** 1 Peter Grünberg Institute 9, Forschungszentrum Jülich GmbH, 52425 Jülich, Germany; 2 Jülich Aachen Research Alliance, Fundamentals of Future Information Technology (JARA-FIT), 52425 Jülich, Germany; 3 Peter Grünberg Institute 2, Forschungszentrum Jülich GmbH, 52425 Jülich, Germany

## Abstract

We study the impact of the direction of magnetic flux on the electron motion in GaAs/InAs core/shell nanowires. At small tilt angles, when the magnetic field is aligned nearly parallel to the nanowire axis, we observe Aharonov–Bohm type *h*/*e* flux periodic magnetoconductance oscillations. These are attributed to transport via angular momentum states, formed by electron waves within the InAs shell. With increasing tilt of the nanowire in the magnetic field, the flux periodic magnetoconductance oscillations disappear. Universal conductance fluctuations are observed for all tilt angles, however with increasing amplitudes for large tilt angles. We record this evolution of the electron propagation from a circling motion around the core to a diffusive transport through scattering loops and give explanations for the observed different transport regimes separated by the magnetic field orientation.

Engineering of the bandgap and interfacial bandstructure of radial and axial heterostructure nanowires leads to interesting implementations of nanowires in quantum information technology. Nanowires allow the three dimensional combination of different materials to form controlled geometric and energetic structures such as tubular quantum rings, quantum dots or flux modulated Josephson junctions^1^,^2^,^3^,^4^,^5^,^6^. The GaAs/InAs core/shell structure investigated is such a special heterostructure, as it combines the highly lattice mismatched materials GaAs and InAs in a layered system^7^,^8^,^9^.

The radial GaAs/InAs heterostructure forms a type-I band alignment, where the low bandgap InAs energetically aligns itself central next to the large bandgap of the GaAs core. At the surface of the InAs, there is experimental and theoretical evidence of a surface accumulation layer due to donor-type surface states^10^,^11^,^12^. This results in the formation of a radial quantum well in the InAs shell in which electrons accumulate^13^,^14^,^15^. The core/shell nanowire therefore harbours a well defined ring geometry, ideal for electron interference in quantum transport experiments.

A consequence of the tubular ring geometry of the electron gas in the heterostructure^16^ are Aharonov–Bohm type oscillations in the magnetoconductance^17^,^18^,^19^,^20^, when a magnetic field is aligned parallel to the nanowires axis. These flux periodic oscillations can be explained by transport through angular momentum states, formed by electron waves within the InAs shell which enclose the penetrating magnetic flux through the core^21^,^22^,^23^,^24^. Theoretical models of this system commonly consider transport through the nanowire to be ballistic and the nanowire axis to be aligned perfectly parallel to the external magnetic field direction^25^,^26^. Both criteria are typically not met in experiment.

Many nanowires suffer from an imperfect crystal structure making the transport diffusive and slight misalignments of the nanowire in the magnetic field are common^27^,^28^,^29^. The defect structure of the nanowire leads to random universal conduction fluctuations (UCF) in the magnetoconductance, typically measured in a perpendicularly oriented magnetic field^30^,^31^,^32^. Recently, Jespersen *et al*. made use of the anisotropy of the UCF pattern of InAs nanowires, when the angle between nanowire axis and magnetic field direction was varied, to gain insight in the effective geometry of the electron system^33^.

In contrast to InAs nanowires, GaAs/InAs core/shell nanowires have a well defined tubular configuration of the electron gas in the InAs shell. Moreover, due to the large confinement potential given by the GaAs/InAs heterojunction and the band bending at the surface, the energy spacing of angular momentum states is in the order of a few meV. This is advantageous for the measurement of Aharonov–Bohm like oscillations. However, their observation will also depend on the presence of electrons having a phase coherence length exceeding the circumference of the shell. UCF are expected to occur already for a much smaller phase coherence length in a mesoscopic system like nanowires. Here, we analyse the dependence of the UCF on the tilt angle between nanowire and magnetic field in this well defined tubular electron system and compare the results to the previously reported dependence for InAs nanowires^33^.

## Experimental Details

**Figure 1:**
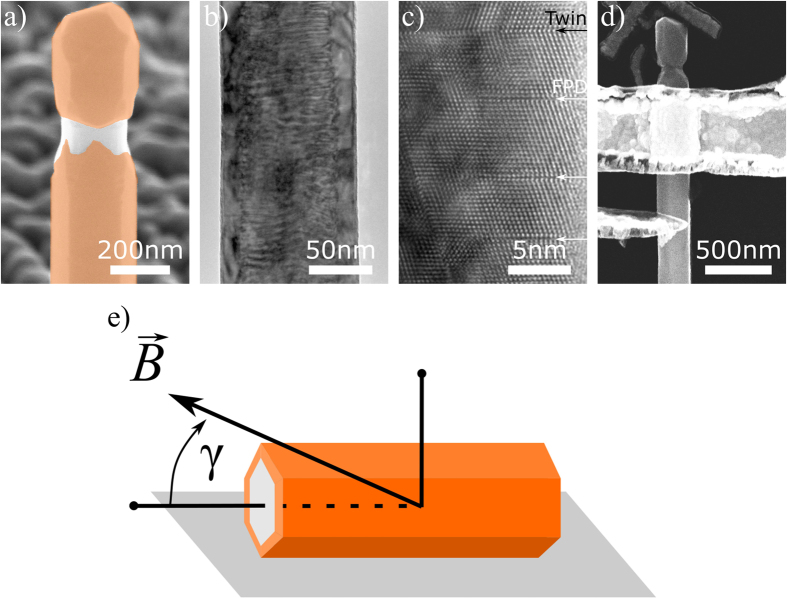
(**a**) Grown GaAs/InAs core/shell nanowire with colourized InAs shell. (**b**) TEM image showing Moiré fringes due to the overlap of the crystal lattices of GaAs and InAs. (**c**) High resolution TEM image of the crystal structure of the InAs shell near the interface to the GaAs core. Frank partial misfit dislocations (FPD) are observed, which induce a stacking fault. Furthermore, a rotational twin adopted from the crystal structure of the core is seen. (**d**) Sample A: GaAs/InAs core/shell nanowire with Ti/Au contacts. (**e**) Definition of the angle of rotation *γ*. For *γ* = 0°, the magnetic field is oriented parallel to the nanowire axis.

GaAs/InAs core/shell nanowires were grown using a self-assisted approach by molecular beam epitaxy, without using a foreign growth catalyst such as gold and without addition of dopants^8^. The growth was carried out on GaAs (111)B growth substrates covered by a thin layer of oxide, which forms nanometer sized pinholes. Within these holes Ga-droplets were created, which act as catalyst for the growth of the GaAs nanowire cores. In the second growth step, the GaAs nanowire cores were overgrown with an InAs shell. Both the GaAs nanowire core as well as the InAs shell have a hexagonal geometry. A typical example of such a GaAs/InAs core/shell nanowire can be seen in Fig. 1(a).

Figure 1(b) shows a transmission electron microscopy (TEM) image of a GaAs/InAs core/shell nanowire. Moiré fringes are observed due to the overlap of the two materials with different lattice constants and indicate an axial strain in the InAs shell^8^. The crystal structure of the nanowires was predominantly zincblende, which is shown in the high resolution TEM image in Fig. 1(c). To relax the axial strain, misfit dislocations have formed which can induce stacking faults in the crystal planes stacking sequence. Furthermore, rotational twins are observed, which are adopted from the crystal structure of the GaAs core. At the top of the nanowires, a small segment of wurzite structure formed, which resulted from the growth during consumption of the Ga-droplet at the end of the growth of the GaAs core. No growth of the InAs shell on the wurzite sidewalls was observed. Therefore, the diameter ∅_*C*_ (given as the distance between two opposite corners of the hexagonal cross section) of the GaAs core and the thickness *t*_*S*_ of the InAs shell could be measured by scanning electron microscopy (SEM). Full details of the growth of GaAs/InAs core/shell nanowires and the crystal structure can be found in refs 8, 9 and 27.

The core/shell nanowires were transferred onto degenerately *n*-doped Si (100) substrates covered by 200 nm thermally grown SiO_2_, which acts as gate dielectric for a backgate. The deposited nanowires were then localized in reference to a pre-patterned marker structure using SEM. Contact leads were defined using electron beam lithography. The contact areas on the wire were cleaned using oxygen plasma and *in situ* Ar^+^ ion milling prior to evaporation of a Ti/Au bilayer and lift-off. The substrates were then diced into smaller pieces and glued and bonded into chip carriers. These pieces were aligned manually within the chip carriers to have a parallel orientation of the nanowire axis and the magnetic field direction in the cryostat later on. Misalignments during this step were checked using SEM and could be determined with about 3° accuracy.

Two nanowires were studied, labelled nanowire A and B (Sample A is shown in Fig. 1(d)). Sample A had a core diameter of 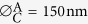 and a shell thickness of 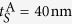. The separation between the contacts used for measuring was *L*^A^ = 260 nm. Sample B had a core diameter of 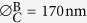, a shell thickness of 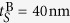 and a contact separation of *L*^B^ = 640 nm.

Measurements were carried out in a He-3 cryostat with a minimum base temperature of 300 mK and in a pumped He-4 cryostat with a variable temperature insert and a minimum base temperature of 1.4 K. Magnetic fields up to 5 T were available in the He-3 cryostat and fields up to 16 T in the latter. The samples were mounted in rotatable sample holders with axis of rotation perpendicular to the magnetic field direction. The tilt angles in the cryostat were controlled via Hall-sensors with about 3° accuracy. Rotation of the samples was possible from *γ*^A^ = 15° to 90° (defined in Fig. 1(e)) for sample A and *γ*^B^ = 5° to 90° for sample B, respectively. Due to small misalignments during the placement of the sample pieces described above, a perfect parallel alignment of the nanowire axis with the direction of the magnetic field was not possible on these samples. In earlier publications, we had shown measurements with parallel alignment, which gave comparable results^18^,^19^. Here, we find that slight misalignments are tolerable for the interpretation of the measurements.

Measurements were performed in two-terminal configuration, biasing with low frequency voltage of typically 50 μV and measuring the current through the nanowire using a low noise *I*/*V*-converter and standard lock-in technique.

## Results

**Figure 2:**
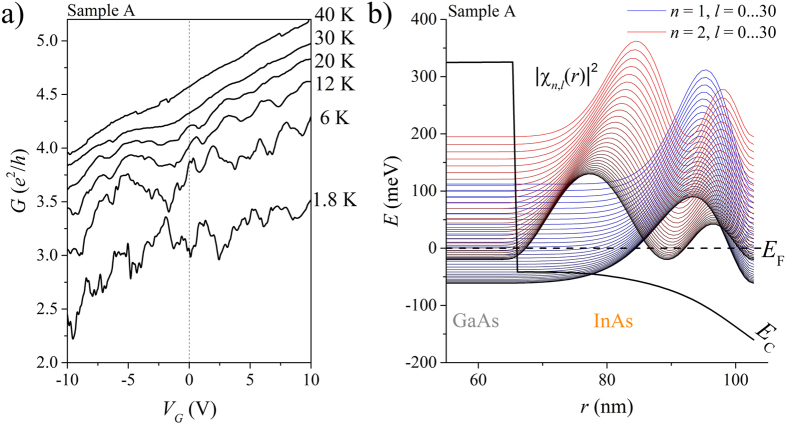
(**a**) Conductance versus applied backgate voltage of sample A at different temperatures. (**b**) Simulation of the angular momentum states in sample A. The conduction band minimum (black line) and the states are plotted versus nanowire radius *r*. The radial probability density |*χ*_*n*,*l*_(*r*)|^2^ (in arbitrary units) of the electrons is shown for radial quantum numbers *n* = 1 (blue) and *n* = 2 (red) as well as for the corresponding angular momentum quantum numbers *l* = 0 … 30. The intersection of a state with the ordinate gives the electron energy of the respective state. The nanowire geometry was approximated by a cylinder of equal cross sectional area as a hexagon.

Figure 2(a) shows the conductance *G* versus backgate voltage *V*_*G*_ of sample A at different temperatures from 40 K down to 1.8 K. The sample shows a decrease in conductance for lowering temperature as expected for a semiconducting nanowire. The remaining conductance of ~3.0 *e*^2^/*h* at the lowest temperature of 1.8 K is a result of the intrinsic accumulation layer within the InAs shell. The positive slope of conductance against backgate voltage indicates the transport in an *n*-type semiconductor. *I*/*V*-characteristics of the samples show over all linear ohmic behaviour at all temperatures.

Below 30 K UCF appear in the gate trace, indicating the onset of diffusive phase-coherent transport in the nanowire^30^. Signs of a stepwise change or plateaus of constant conductance with changing *V*_*G*_ are not observed, which excludes ballistic transport for our sample. The UCF appear as consequence of electron wave packet interference, but only if the sample dimensions are of the same order or smaller than the phase coherence length of the sample. These fluctuations are caused by phase-coherent transport of the electrons through randomly formed loops, which stem from elastic scattering at fixed impurities and defects at low temperatures. The electron waves can interfere constructively or destructively with themselves when traversing through these loops. This results in a fluctuation pattern in the conductance, when an external parameter such as *V*_*G*_ or *B* is changed, as these cause a change in the phase accumulation of the electron waves during passage through the scattering loops^30^,^34^,^35^.

We simulated the electron states in our GaAs/InAs core/shell nanowires for both samples using a Schrödinger–Poisson equation solver to determine the available transport channels^18^. The results for sample A are plotted in Fig. 2(b) (results for sample B are similar). For both samples we find several angular momentum states located beneath the Fermi level *E*_*F*_, which can contribute to the transport. Yet, due to the observation of diffusive transport and lower conductance than expected for the ballistic case, where each channel contributes with 2 *e*^2^/*h* to the conductance, the transparency of each transport channel must be low. This is attributed to the large amount of stacking faults and misfit dislocations in our core/shell system^8^, which effectively reduce the mean free path of the electrons to a few nanometers.

**Figure 3:**
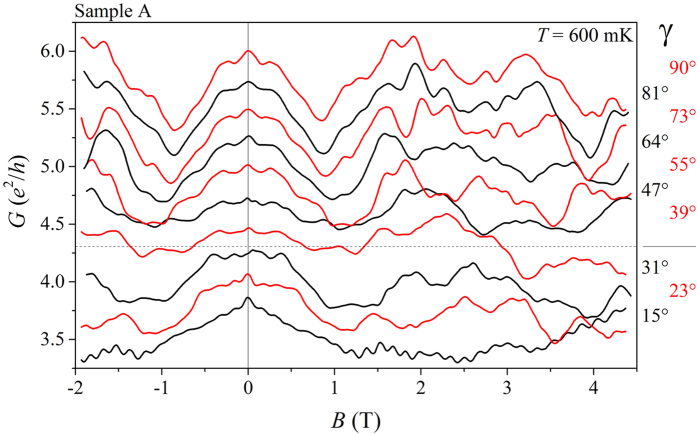
Magnetoconductance of nanowire A at different angles *γ* with respect to the magnetic field direction as defined in Fig. 1(e). The curves are offset by Δ*G* = 0.25 *e*^2^/*h* for clarity. A dotted guideline is provided to help separating the tilt ranges described in the text. For small tilt angles Aharonov–Bohm type oscillations are clearly visible, while for larger tilt angles an UCF pattern dominates the signal.

Figure 3 shows the magnetoconductance of sample A at selected tilt angles from a nearly parallel orientation of the nanowire axis with the magnetic field (*γ*^A^ = 15°) to a perpendicular oriented field (*γ*^A^ = 90°). The measured curves are all symmetric around zero magnetic field, therefore fulfilling the Onsager symmetry relation expected for two terminal transport. We distinguish two different tilt ranges (separated by the horizontal guideline in Fig. 3), where a clear change of the magnetoconductance signal is observed. The magnetoconductance of sample B is showing a similar behaviour as sample A and is given in Fig. S1 in the supplementary information.

**Figure 4:**
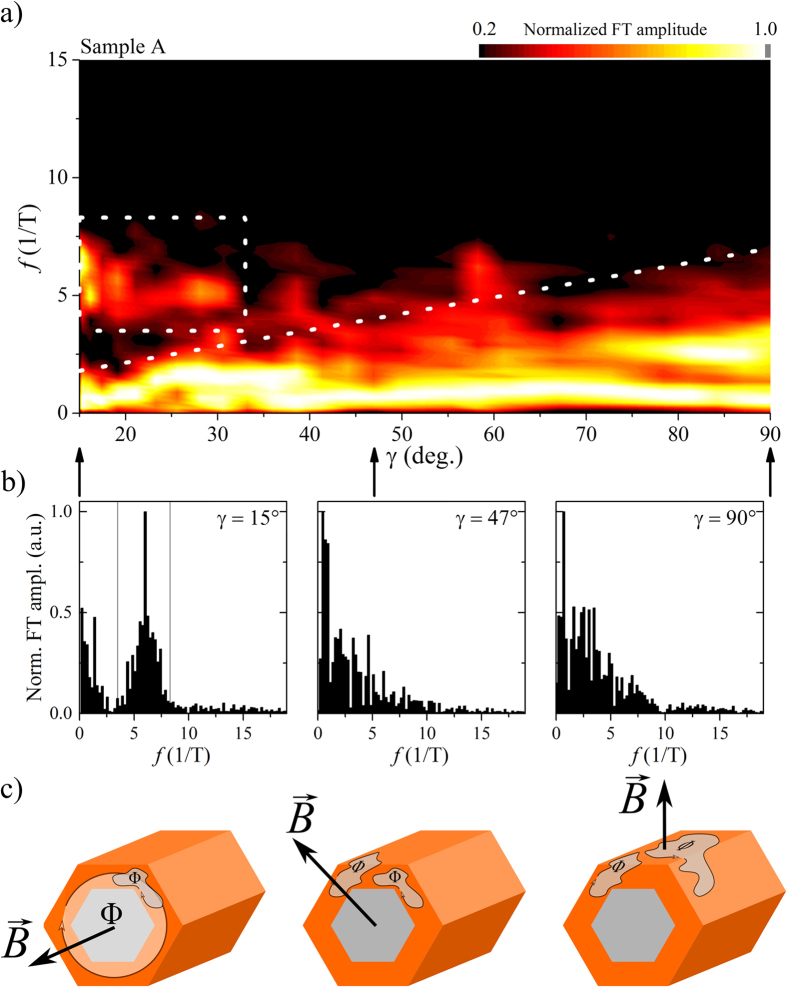
(**a**) Normalized and smoothed Fourier transformation amplitudes of the differentiated magnetoconductance measurements of Fig. 3. Three profile cuts for *γ*^A^ = 15°, 47° and 90° are given in (**b**). The dotted box for *γ* ≤ 31° marks the expected frequency range for an electron enclosing magnetic flux quanta Φ_0_ = *h*/*e* while moving on the very outermost or innermost perimeter of the InAs shell. The illustrations in (**c**) show potential closed electron paths, either caused by the nanowire core/shell geometry or by defect scattering, which each enclose magnetic flux Φ at different magnetic field alignments. The former results in Aharonov–Bohm type magnetoconductance oscillations, the latter in UCF. The size and multitude of such closed loops determines the main components of the Fourier amplitudes. With increasing tilt the background UCF become the most prominent feature of the spectrum, highlighted by a dotted guideline.

At lowest angles *γ*^A^ ≤ 31° we observe clearly visible periodic magnetoconductance oscillations with an average period of Δ*B*^A^ = 170(3) mT over the full range of magnetic fields on top of a slowly varying background. Additional measurements on sample A as well as on sample B have shown, that these periodic oscillations prevail up to 14 T without notable decrease in amplitude at high magnetic fields. By differentiating the data with respect to the magnetic field *B*, we calculate the corresponding Fourier transformation (FT) of the measurement without the constant conductance offset and focus on the low and high frequency components of the signal. The results for all measurements made on sample A are shown in Fig. 4 (see Fig. S2 in the supplementary information for sample B). The results were normalized to compare the main frequency contributions between all tilt angles. At lowest angles *γ*^A^ ≤ 31°, we observe two distinct frequency components with nonzero contribution to the Fourier spectrum, one reaching from *f* = 0 to 3.0 T^−1^ and the second ranging around a clear peak at a frequency of about *f* = 6.0 T^−1^ (marked by the dotted box in Fig. 4(a)).

The higher frequency components are attributed to an Aharonov–Bohm type oscillation by an electron enclosing a magnetic flux Φ = Δ*B* ⋅ *A* = *h*/*e* while moving around the GaAs core within the InAs shell^18^,^19^. The horizontal boundaries of the dotted box in Fig. 4(a) mark the theoretically expected frequency range for encircling the core at the very innermost and outermost perimeter of the shell (see also the profile cut for *γ*^A^ = 15° in Fig. 4(b)). The measured frequencies correspond well to this expectation and also to the simulated probability density of the closed-ring angular momentum states from Fig. 2(b). The phase coherence length in the cross sectional plane and therefore perpendicular to the transport direction and the stacking faults in axial direction must hence be larger than the circumference, so that an angular momentum state can form. Earlier measurements on pure InAs nanotubes based on the GaAs/InAs core/shell nanowire have also shown the same flux periodic oscillation, so transport through the high bandgap GaAs can be excluded^16^.

The lower frequency components in the range *f* = 0 ... 3.0 T^−1^ are clearly separated and discernible from the Aharonov–Bohm type oscillations and rapidly decrease to higher frequencies. These low frequency components are caused by the slowly varying background underneath the periodic oscillations. As there is no transport within the GaAs core^16^ and magnetic flux enclosure by an electron encircling the core causes frequencies of at least 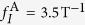 and above (given by the boundary of the box in Fig. 4(a)), the low frequency oscillations must be caused by UCF from flux enclosure within loops formed by randomly distributed elastic scattering centres inside the InAs shell cross section. Transport at such low angles is therefore a superposition of the contributions from transport through angular momentum states, where flux is enclosed in the nanowire cross section, next to a diffusive electron motion where flux is enclosed in scattering loops in the shells cross sectional area. This situation is illustrated in the left illustration of Fig. 4(c).

With increasing angle between nanowire axis and magnetic field direction, we observe a decrease in the Fourier amplitude of the Aharonov–Bohm type oscillations while the UCF background gains additional higher frequency components, illustrated by the dotted guideline in Fig. 4(a). At roughly *γ*^A^ ≈ 31° the flux periodic oscillations disappear and can no longer be separated from the UCF background. This effect is due to the reduced magnetic flux through the nanowire cross section, relevant for the formation of the coherent angular momentum states. When tilting the magnetic field away from the nanowire axis, the axial and angular motion of the electrons can no longer be separated^28^,^29^. The electrons are now forced to move on a plane tilted against the cross section of the nanowire. Their phase coherence length can be reduced in this direction as they are now also propagating through the crystal planes of the stacking faults^36^. Comparable InAs nanowires measured in a perpendicular oriented magnetic field have phase coherence lengths of a few hundred nanometers^32^ and hence lower than needed for a coherent motion around the circumference of our core/shell nanowire. At large angles a coherent closed-ring angular momentum state cannot form and the *h*/*e* flux periodic oscillation disappears.

**Figure 5:**
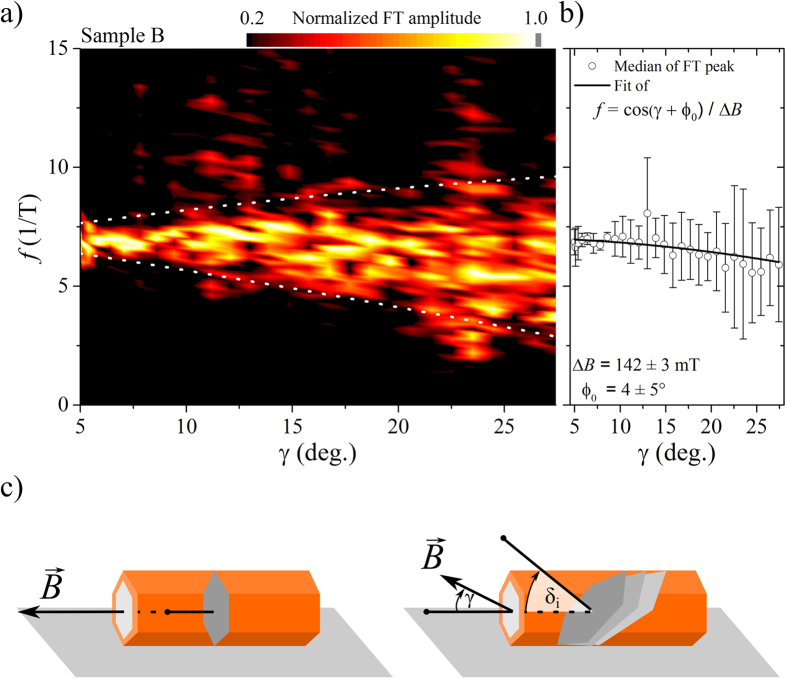
(**a**) Normalized amplitudes of the Fourier transformation of the magnetoconductance of sample B at low tilt angles and with high resolution to follow the evolution of the Aharonov–Bohm type oscillation pattern. The white dotted lines indicate the bounds of the frequency spreading with increased tilt proportional to sin (*γ*) as given by equation 3, caused by loop projections tilted in field as described in the text. (**b**) Medians of the frequency distribution from (**a**) determined from fitting a Gauss normal distribution on the data. The data is well described by a cosine dependence in tilt angle *γ* as described in the text. (**c**) The illustrations show contributions of different areas picking up magnetic flux. At parallel field (*γ* = 0°) mainly the perpendicular flux through the cross section determines the oscillations frequency. With increasing tilt, slightly tilted projections can also contribute to the oscillation pattern causing a spread of the observed frequencies.

To further analyse the evolution of the Aharonov–Bohm type oscillations with increasing tilt, we measured the magnetoconductance of sample B at low angles *γ*^B^ = 5° to 30° with higher resolution in angle and magnetic field. In Fig. 5(a) we have plotted the amplitudes of the Fourier spectrum of this measurement for these small tilt angles. High pass filtering was used on the differentiated data prior to Fourier transformation to remove the low frequency background and isolate the oscillation. The centre frequency of the flux periodic oscillations is moving to lower frequencies, as can be seen from Fig. 5(b) where we have fitted a Gauss normal distribution to the Fourier transformation of Fig. 5(a) to determine the frequencies median. Furthermore, the distribution of the frequency components becomes broader the further we tilt the nanowire away from the parallel orientation to the magnetic field.

We can model this behaviour via a simple approach. We assume, that with increasing tilt of the magnetic field additional tilted projections of the paths encircling the core become available to enclose flux (tilted by *δ*_*i*_, see the illustrations in Fig. 5(c)). The angle *γ* takes into account the deviation of the magnetic field towards the cross sections normal, whereas *δ*_*i*_ is an additional tilt with respect to the already misaligned magnetic field direction. The contribution of the tilted paths to the frequency spectrum is then given by their geometric projection on the nanowires cross section. These areas enclose the flux


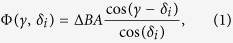


where *A* is the cross sectional area of the nanowire for the non-tilted magnetic field. We can see, that in the parallel case *γ* = 0°, these tilted paths have no effect and only the regular Aharonov–Bohm oscillation Δ*BA* = *h*/*e* remains, which corresponds to the case of separated axial and angular motion. Only for *γ* > 0°, these paths enclose a different flux than the nanowires cross sectional area. We assume a uniform distribution *P*(*δ*_*i*_) = 1/2*δ*_Max_ of these tilted paths, where *δ*_Max_ is the angle of the maximal tilted projection allowed, which can be estimated to be the angle where a tilted path connects the contacts *δ*_Max_ ≤ arctan(*L*/2*r*). The central frequency *f* = 1/Δ*B* of the distribution, for which a flux quantum *h*/*e* is enclosed, should then follow via averaging of the magnetic flux through all areas


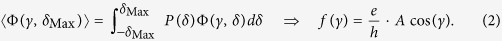


This cosine dependence is indicated by the fit in Fig. 5(b) (with an additional phase *ϕ*_0_ for an angular offset) and describes the data very well. Furthermore, when we can analogously calculate the variance assuming a uniform distribution of the tilted paths. The frequency spectrum is then supposed to spread as


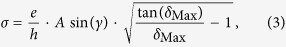


which is plotted as white dotted line in Fig. 5(a). The spread is proportional to sin(*γ*) and describes the observed Fourier spectrum reasonably well. The tilted trajectories are therefore causing a mixture of the angular and the axial motion of the electrons in the nanowire, until the circumference can no longer be encircled phase coherently and the oscillations disappears underneath the UCF background.

When we increase the angle between nanowire axis and magnetic field past the range of the Aharonov–Bohm type oscillations (*γ*^A^ > 31°) additional higher frequencies appear in the Fourier spectrum of the UCF, highlighted by the dotted line in Fig. 4(a). Furthermore, the UCF pattern stabilizes at large angles so that positions of maxima and minima in the curves can be traced (see Fig. 3). This results from the magnetic flux which now penetrates larger parts of the nanowire sidewall. The flux is therefore enclosed by scattering loop areas in axial direction of the nanowire, which can be much larger than the loops forming in plane of the shell cross section, as illustrated in the central and right image of Fig. 4(c). With larger tilt angle, more and more scattering loops of different size enclose the magnetic flux and contribute to the UCF. Most of these loops are small as the main emphasis of the Fourier spectrum lies at low frequencies. Larger loop areas require larger phase coherence lengths to be enclosed coherently and only contribute smaller amounts.

**Figure 6:**
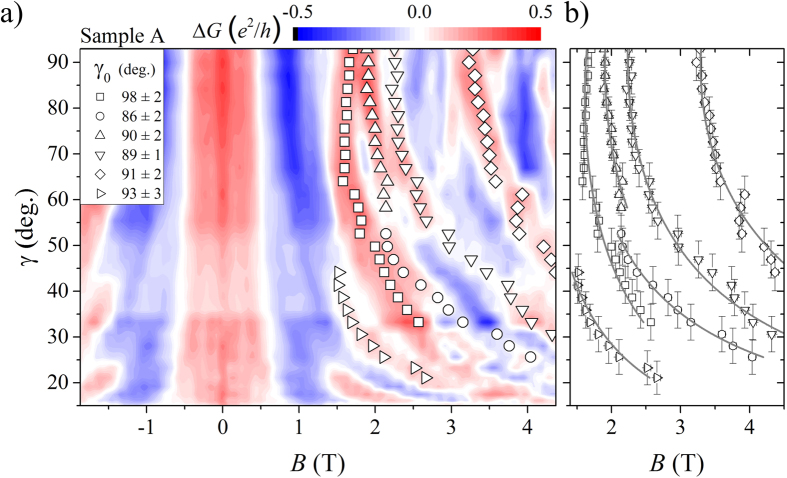
(**a**) Differential conductance in colour code versus tilt angle *γ* and magnetic field *B* of sample A. The subtracted background was determined by adjacent average smoothing over the whole measurement of Fig. 3. All the maxima and minima of the curves bend to higher magnetic fields with decreasing angle between nanowire axis and magnetic field direction, which indicates a maximum of flux enclosure within the InAs shell at perpendicular aligned magnetic field. Several maxima positions are followed for different tilt angles and plotted as symbols. Their course of progression with decreasing tilt is fitted with equation 4 shown as grey solid lines in (**b**)^33^.

In Fig. 6(a) we have plotted the UCF maxima and minima in colour code for sample A by subtracting a background determined by smoothing over the whole curve for each trace at each tilt angle of Fig. 3 (the colour coded UCF pattern of sample B is shown in Fig. S3 in the supplementary information). The typical UCF oscillation amplitude is of order 0.1 *e*^2^/*h* with maximum amplitudes of Δ*G* ≈ 0.4 *e*^2^/*h* in agreement with previous measurements on homogeneous nanowires^31^,^32^. Maxima and minima positions bend symmetrically to higher magnetic fields, when we move from *γ* = 90° to lower tilt angles. This bending of the peak positions of an UCF pattern of similar amplitude has also been observed for InAs nanowires^33^. The bending of the maxima and minima is attributed to a varying flux enclosure within single scattering loops at different angles. It can be used to find the angle *γ*_0_, where the magnetic field enclosure of most scattering loops becomes maximal.

To quantify this effect, Jespersen *et al*.^33^ have assumed, that the flux enclosure in the scattering loops follows


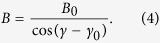


This equation states, that every scattering loop contributes to the magnetoconductance at different tilt angles *γ* dependent on the projection of its area, but with a maximum contribution at *γ*_0_. In Fig. 6(a), we have marked the peak positions at different tilt angles of six UCF peaks for sample A (five UCF peaks were measured for sample B; see Fig. S3 in the supplementary information). These positions have been fitted with Eq. 4 as seen in Fig. 6(b) and its results are stated in the inset of Fig. 6(a). The determined mean angles of maximum flux enclosure are 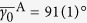 for sample A and 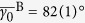 for sample B, respectively.

These results state, that in a nanowire with a ring-like surface accumulation layer and without bulk contribution from the core, the maximum flux enclosure is still found for a perpendicular oriented magnetic field, just as in the bulk InAs nanowires from ref. 33. The scattering loops in the nanowire sidewalls are therefore the main contributors to the observed magnetoconductance oscillations at large tilt angles. Yet, at small tilt angles flux enclosure and transport in angular momentum states can additionally be resolved in the magnetoconductance, as the otherwise dominating UCF have a longer oscillation period. Only at larger angles, an encircling of the core is no longer possible phase coherently and UCF become the only dominant feature of the magnetoconductance.

## Conclusion

We have measured the magnetoconductance of GaAs/InAs core/shell nanowires at different tilt angles between nanowire axis and the external magnetic field direction to show the great potential angle resolved measurements offer to resolve electron motion in nanowires. We could observe the evolution from Aharonov–Bohm type flux-periodic oscillations on top of a slowly varying background at close to parallel magnetic field orientation to pronounced UCF at perpendicular oriented field direction. At small tilt angles two types of oscillations are observed. Aharonov–Bohm type oscillations are obtained due to the interference of electron waves with a phase coherence length longer than the circumference of the shell. Second, UCF oscillations formed within small scattering loops inside the thickness of the InAs shell are present. Typically the amplitude of Aharonov–Bohm like oscillations is smaller than for UCF since the number of electrons having a phase coherence length larger than the circumference of the nanowire will be substantially smaller than those with sufficient coherence length for small scattering loops. A distribution model of tilted electron trajectories was developed to explain the reduced periodicity and spread of the observed frequency components at small angles. At larger tilts, the Aharonov–Bohm type oscillations disappear as the electrons are unable to coherently encircle the GaAs core. Therefore, most of the magnetic flux is penetrating scattering loops formed by diffusively propagating electrons in the nanowires sidewalls. For large angles, analysis and modelling of the UCF showed that the position of maximal flux enclosure by the scattering loops is found close to perpendicular oriented magnetic field. In general, for our core/shell nanowires the dependence of the UCF characteristics on the tilt angle between the magnetic field and the nanowire axis is similar to that obtained for InAs nanowires. This might be attributed to the thickness of the InAs shell, which gives sufficient room for small scattering loops. However, a small shell thickness will lead to even lower frequencies of the UCF, separating the frequencies of the Aharonov–Bohm type oscillations and the UCF even further. Thus we conclude, that the measurement of the angular dependence of UCF characteristics requires careful analysis of the size of the scattering loops with respect to the nanowire diameter, in order to conclude on the configurations of the electron gas.

## Additional Information

**How to cite this article**: Haas, F. *et al*. Angle-dependent magnetotransport in GaAs/InAs core/shell nanowires. *Sci. Rep*. **6**, 24573; doi: 10.1038/srep24573 (2016).

## Supplementary Material

Supplementary Information
