# Supplementary material for: Angle-dependent magnetotransport in GaAs/InAs core/shell nanowires
Source: Sci Rep. 2016 Apr 19;6:24573. doi: 10.1038/srep24573 (PMC4835758; doi:10.1038/srep24573)
Supplement: Supplementary Information [file srep24573-s1.pdf]

Supplementary Information: Angle-dependent magnetotransport in GaAs/InAs core/shell nanowires

Fabian Haas[1,3,*], Tobias Wenz[1,3], Patrick Zellekens[1,3], Nataliya Demarina[2,3], Torsten Rieger[1,3], Mihail Lepsa[1,3], Detlev Grützmacher[1,3], Hans Lüth[1,3], and Thomas Schäpers[1,3,+]

[1]Peter Grünberg Institute 9, Forschungszentrum Jülich GmbH, 52425 Jülich, Germany
[2]Peter Grünberg Institute 2, Forschungszentrum Jülich GmbH, 52425 Jülich, Germany
[3]Jülich Aachen Research Alliance, Fundamentals of Future Information Technology (JARA-FIT), 52425 Jülich, Germany
[*]f.haas@fz-juelich.de
[+]th.schaepers@fz-juelich.de

ABSTRACT

Measurements on sample B:

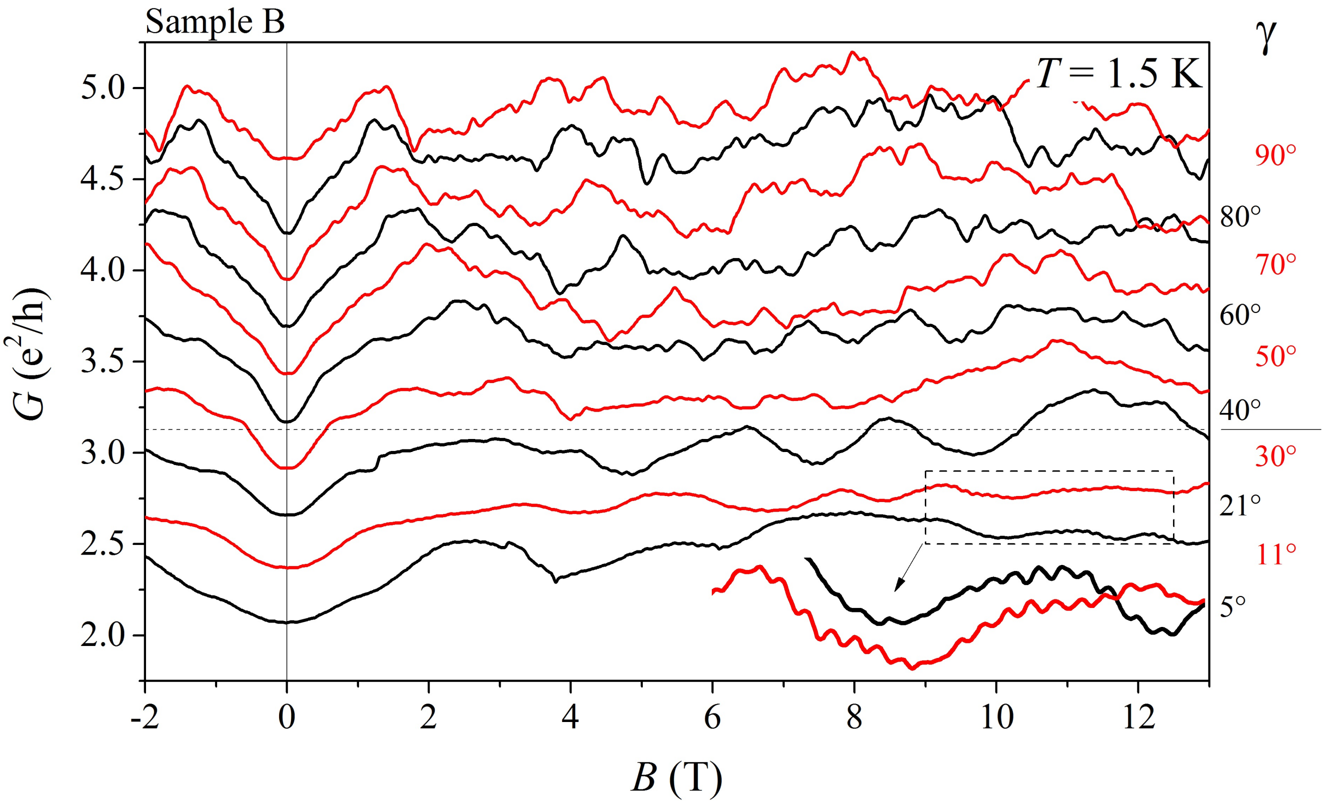

Figure S1. Magnetoconductance of nanowire B at different selected tilt angles γ with respect to the magnetic field direction. The curves are offset by $\Delta G = 0.25\, e^2/h$ for clarity. The inset shows the periodic Aharonov–Bohm type oscillations in the marked box between $B = 9\,\mathrm{T}$ to $12.5\,\mathrm{T}$ for low angles, as their amplitude is too low to be recognized on the larger scale picture. Note, that the curves in the inset are not offset.

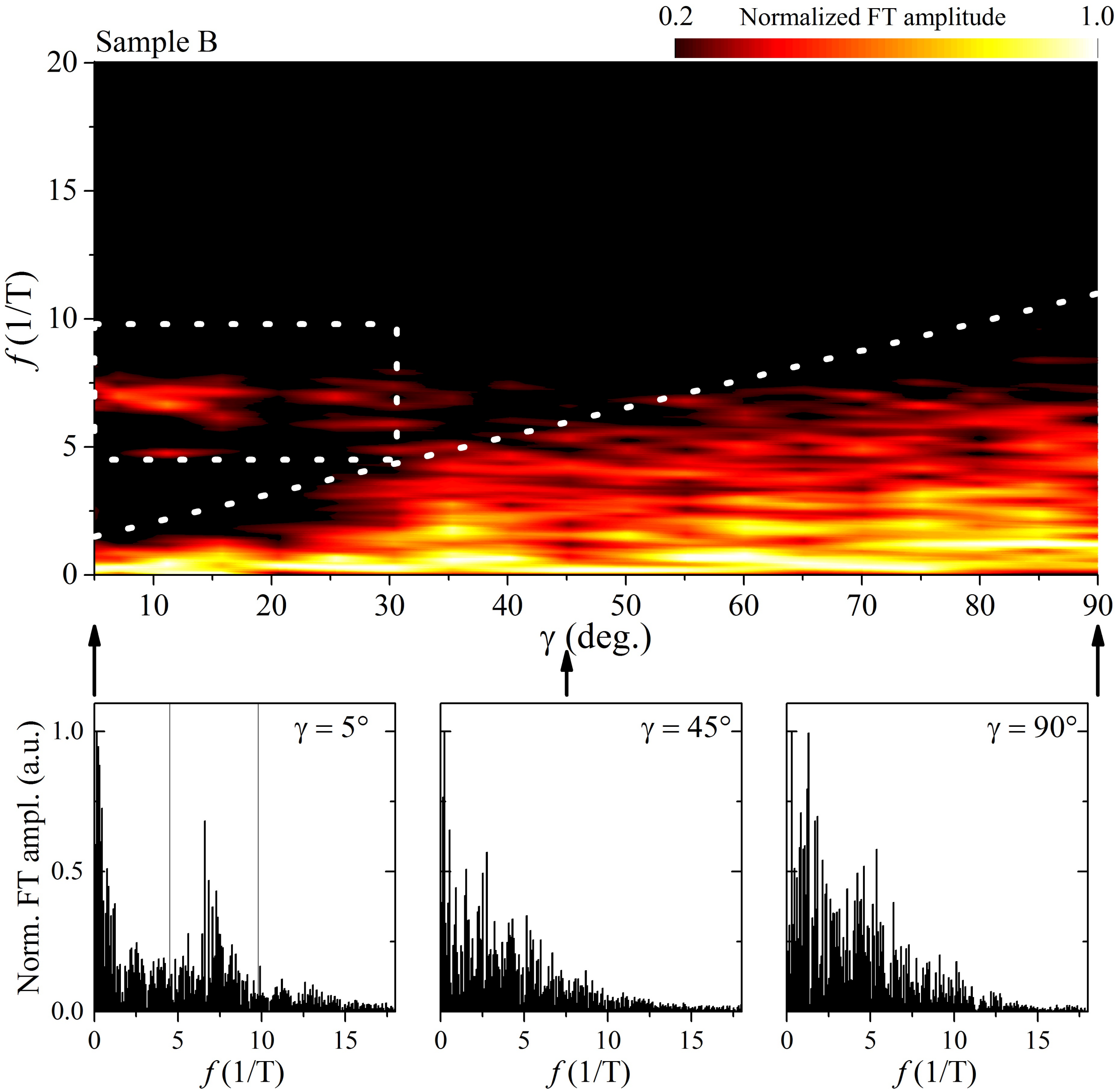

Figure S2. Normalized and smoothed Fourier transformation amplitudes of the differentiated magnetoconductance measurements of Fig. S1 for sample B. Three profile cuts for $\gamma^{A} = 5°$, $45°$ and $90°$ are given. The horizontal boundary of the dotted box marks the expected frequency range for an electron enclosing magnetic flux quanta $\Phi_0 = h/e$ while moving on the very outermost or innermost perimeter of the InAs shell of nanowire B. With increasing tilt the background UCF become the most prominent feature of the spectrum, highlighted by a dotted guideline.

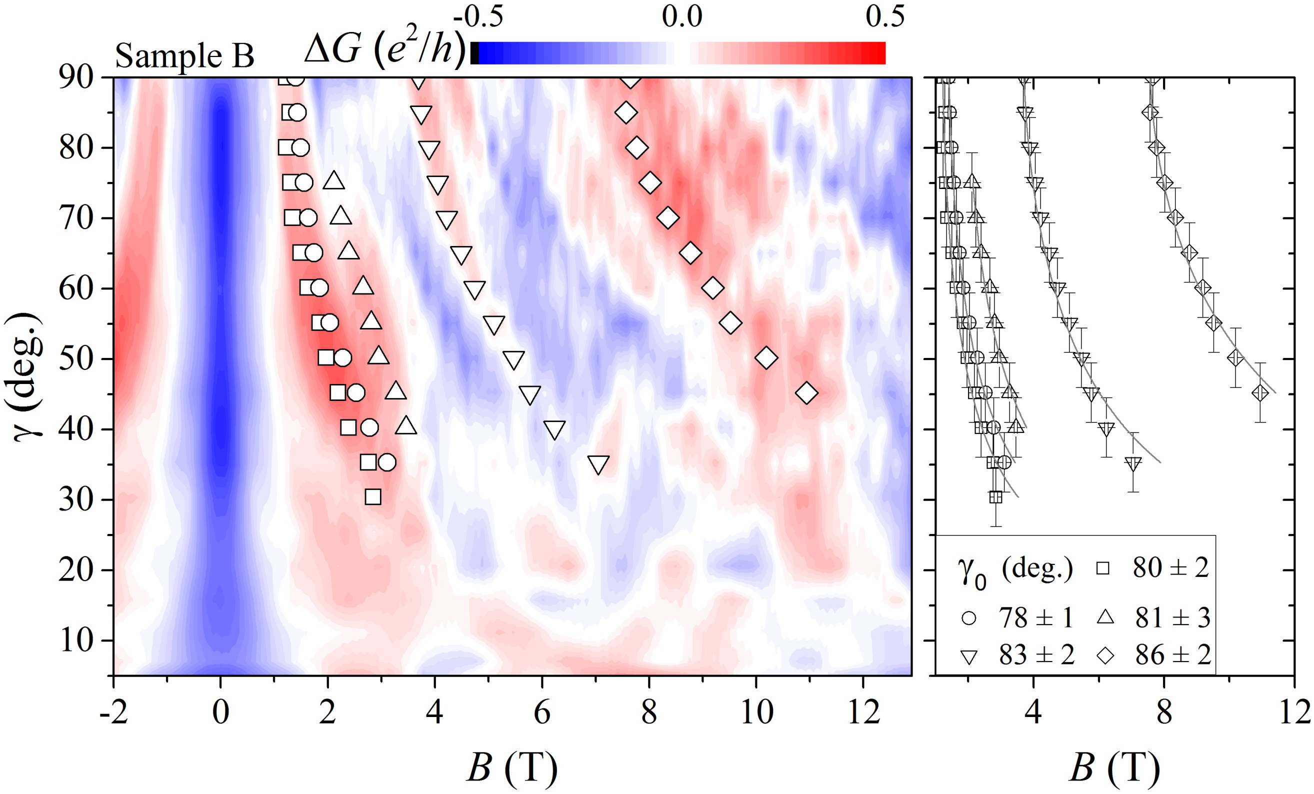

Figure S3. Differential conductance in colour code versus tilt angle γ and magnetic field B of sample B. The subtracted background was determined by adjacent average smoothing over the whole measurement of Fig. S1. All the maxima and minima of the curves bend to higher magnetic fields with decreasing angle between nanowire axis and magnetic field direction, which indicates a maximum of flux enclosure within the InAs shell at perpendicular aligned magnetic field. Five maxima positions are followed for different tilt angles and plotted as symbols. Their course of progression with decreasing tilt is fitted with the equation $B = B_0/\cos(\gamma - \gamma_0)$ shown as grey solid lines in the right picture.
